# FAPI-04 Uptake in Healthy Tissues of Cancer Patients in ^68^Ga-FAPI-04 PET/CT Imaging

**DOI:** 10.1155/2021/9750080

**Published:** 2021-11-23

**Authors:** Cihan Gündoğan, Yunus Güzel, Canan Can, İhsan Kaplan, Halil Kömek

**Affiliations:** Department of Nuclear Medicine, Gazi Yaşargil Training and Research Hospital, University of Health Sciences, Üçkuyular Mah. Elazığ yolu üzeri 10. Km, 21070, Kayapınar, Diyarbakır, Turkey

## Abstract

**Objective:**

The aim of this study is to investigate the uptake of ^68^Ga-FAPI-04 in normal tissues and calculate standardized uptake values (SUVs) for various organs in the body.

**Methods:**

A total of 49 patients who underwent ^68^Ga-FAPI-04 PET/CT were included in our study. The following organs were identified on CT images: brain, parotid, and submandibular glands, palatine tonsils, thyroid, lymph nodes (if present), breasts, lungs, thymus, left ventricle walls, mediastinal blood pool, vertebral bone marrow, liver, spleen, pancreas, stomach, small and large intestines, adrenal glands, kidneys, uterus, testes, and prostate. Median, minimum, and maximum values (max) and average (avg) values of standard uptake value (SUV) of tissues and organs were calculated.

**Results:**

The accumulation of ^68^Ga-FAPI in normal organs showed variations. The cerebral/cerebellar cortex exhibited no ^68^Ga-FAPI uptake, while the scalp showed low uptake. Low uptake was also observed in the lung parenchyma, esophagus, left ventricle walls, nipple, and glandular breast tissue. In the abdominopelvic area, the pancreas exhibited low uptake, which was higher in the tail region. Low uptake was observed in the renal cortex. Intense ^68^Ga-FAPI uptake was observed throughout the uterus, which was higher in the corpus. There was no uptake of ^68^Ga-FAPI in the bone cortex and medulla.

**Conclusion:**

We determined the physiological uptake and SUVmax of FAPI-04 in different tissues and organs and created a guide for researchers.

## 1. Introduction

Cancer is expected to be the leading cause of death and the main obstacle against improvements in life expectancy in every country of the world in the 21st century. Only in 2018, the estimated number of new cancer cases was 18.1 million and cancer deaths was 9.6 million [1]. Fast and accurate diagnosis and staging of cancer patients will improve management options and increase expected survival. Therefore, improvements in imaging techniques are more popular in recent research.

Positron emission tomography/computed tomography (PET/CT) has been widely used in the diagnosis, staging, and evaluation of treatment response in various types of cancer and even nonmalignant diseases worldwide since its introduction. The most commonly used PET radiopharmaceutical is 18F-fluorodeoxyglucose (FDG). The sensitivity and specificity of PET/CT is poor in certain types of cancer including prostate cancer, neuroendocrine tumors, mucinous tumors, and signet-ring cell tumors, which exhibit low FDG affinity [2]. Recently, with the development of gallium-68-labeled compounds, novel radiotracers including prostate specific membrane antigen (PSMA) and DOTA-DPhe1, Tyr3-octreotate (DOTATATE) have been replacing FDG in these tumors. However, these agents may also remain inadequate in certain settings, with their false positivity and negativities [3, 4].

Fibroblast activation protein (FAP) was first described in 1986 as a cell surface antigen [5]. It is highly expressed on the reactive stromal fibroblasts of many epithelial carcinomas, soft tissue sarcomas, granulation tissue, and certain fetal mesenchymal fibroblasts [6]. FAP is a 97-kDa type II membrane-bound glycoprotein with serine protease activity. It is a member of the prolyl peptidase family. Its closest relative is dipeptidyl peptidase IV (DPPIV and CD26) from the same family, and these two proteins share 70% amino acid sequence homology [7]. FAP has a total of 760 amino acids, with 1–4 composing the intracellular domain, 5–25 composing the transmembrane domain, and 26–760 composing the extracellular domain. The circulating alpha2-antiplasmin cleaving enzyme (APCE) results from posttranslational cleavage and is, thus, created from the extracellular portion of FAP [8].

In histopathologic studies, FAP-positive cancer-associated fibroblasts were found in over 90% of epithelial tumors, which makes FAP a potential target for imaging and therapy in a large variety of malignancies [9]. Quinoline-based inhibitors labeled with diagnostic and therapeutic radioisotopes via the chelator DOTA were developed by the Heidelberg group [10, 11]. Recently, ^68^Ga-DOTA-labelled-FAP inhibitors (FAPI) PET/CT have been used in several cancers [11–13]. Also, a number of studies have reported that nonmalignant may also exhibit FAPI uptake [14–18]. Dynamic PET/CT imaging of ^68^Ga-FAPI-04 study, which was performed on 6 patients with 3 healthy Chinese subjects and 3 patients with lung cancer, has recently been presented, and a research need arose in a larger population due to the low number of patients [19]. Therefore, a need to thoroughly determine the physiologic distribution of ^68^Ga-FAPI in normal tissues and calculate the standardized uptake values (SUVs) for various organs in the body has arisen.

## 2. Materials and Methods

### 2.1. Patient Selection

A total of 49 patients who participated in the ^68^Ga-FAPI-04 PET/CT studies carried out in our clinic were evaluated. These randomly selected patients underwent ^68^Ga-FAPI PET/CT study because imaging with FDG, PSMA, or DOTATATE was inconsistent with clinical or laboratory findings and did not yield satisfactory results. Thirty-four patients were women and 15 were men, with a median age of 51 years (17–86 years). Among these, 16 patients had breast cancer, 11 had gastric cancer, 4 had colorectal cancer, 3 had ovarian cancer, 2 had pancreatic cancer, 3 had neuroendocrine tumor, 2 had lung cancer, 2 had Ewing's sarcoma, 2 had duodenal cancer, 1 had germ-cell tumor, 1 had lymphoma, 1 had chondrosarcoma, and 1 had medullary thyroid cancer. Approval from the institutional review board of Gazi Yaşargil Training and Research Hospital Ethics Committee (2021/652) was obtained prior to the start of the study.

### 2.2. Labelling Technique: Radiopharmaceutical Synthesis

The radiotracer was synthesized by a fully automated, Good Manufacturing Practice- (GMP-) compliant procedure using a standardized labeling sequence with a (^68^Ge)/(^68^Ga) generator (iThemba. Labs, S. A), 25 *μ*g (15 nmol) of FAPI-04, and a GRP® module (SCINTOMICS GmbH, Fürstenfel dbruck, Germany) equipped with a disposable single-use cassette kit (ABX, Radeberg, Germany) as described previously in the literature [10, 20, 21]. Briefly, radiolabeling was performed by adjusting a mixture of 25 *µ*gr FAPI-04, 3 mL Hepes solution, and 1.5 mL ^68^Ga-solution (0.5–0.6 GBq in 5 M-1.5 mL NaCl_3_ to pH 3.5). The mixture was passed through a C18 cartridge after 20 minutes of heating at 100 °C. 1 mL of ethanol and 1 mL of water were eluted from the cartridge, and 14 mL of phosphate buffer solution was added. The pH was adjusted to pH 7 by dilution with phosphate buffer solution.

The labeling efficiency and radiochemical purity were determined using radio-TLC and radio-HPLC (HPLC Agilent 1260 Infinity liquid chromatography system (Agilent Technologies, Palo Alto, CA, USA)). The radiochemical purities of ^68^Ga-labeled FAPI-04 conjugates were ≥95%. Following HPLC-based quality control, 2 MBq/kg of (^68^ Ga) FAPI-04 was injected.

### 2.3. PET/CT Protocol

All images were obtained using the Discovery IQ 4 ring 20 cm axial FOV PET/CT (GE Healthcare, Milwaukee, WI, USA). After the injection, the whole-body images were taken from the vertex to middle of the thigh at the 1^st^ hour. After CT images (CT parameters: 120 kV, 80 mAs/slice, 700 mm trans axial FOV, no gap, 64 × 0.625 mm collimation, pitch 1.4. 0.5 s rotation time, 3.3 mm slice thickness, and 512×512 matrix), PET images (PET parameters: 3D FOV 20 cm, ordered subset expectation-maximization algorithm (OSEM) 5 iterations/12 subset, and full width at half maximum (FWHM) 3 mm) were taken at the bedside at 2.5 min in the same position to include the same regions. Point HD and sharp IR programs were used for postreconstruction assessments. All patients without contraindications were given IV contrast at a dose of 1.5 ml/kg, 50 seconds before the CT imaging. Attenuation corrected emission images were obtained using contrast or noncontrast CT data. All patients were asked to drink water before PET/CT imaging and urinated immediately before imaging.

### 2.4. Image Analysis

All ^68^Ga-FAPI-04 PET/CT were reviewed on AW 4.7 (Advantage Workstation software version 4.7, GE Healthcare, Milwaukee, WI, USA) by two experienced nuclear medicine physicians. For each study, the patient's height and weight, administered activity, and uptake time were noted. The following organs were identified on the CT images: the brain(cerebrum/cerebellum), parotid and submandibular glands, palatine tonsils, thyroid, lymph nodes (if present), breasts, lungs, thymus, left ventricle walls, mediastinal blood pool, vertebral bone marrow, liver, spleen, pancreas (tail/corpus), stomach, small and large intestines, adrenal glands, kidneys, uterus, testes, prostate, muscles, and bones. Primary tumor SUVmax values, if any, were also measured. Healthy tissue SUVmax was calculated from the tumor-free areas which were made from areas not suggestive of primary tumor or metastasis on ^68^Ga-FAPI-04 PET/CT. Areas of interest were drawn from the right-side tissues of the body, except for metastases in symmetrical tissues. Volume of interest (VOI) was drawn over three consecutive slices on the PET images centered around the maximum voxel value for the abovementioned organs, and the maximum and average (avg) values of the SUVs in the VOIs were recorded. The VOIs were always placed within the limits of the activity distribution to minimize the partial volume effect. VOIs were drawn as 1 cm in small tissues and 2 cm in large organs including the lungs and liver.

### 2.5. Statistical Analysis

SPSS version 25.0 (Statistical Package for Social Sciences; IBM SPSS Corp; Armonk, NY, USA) was used for statistical analyses. Median, mean, minimum, and maximum values for SUVmax and SUVavg of tissues and organs were calculated.

## 3. Results

**Figure 1:**
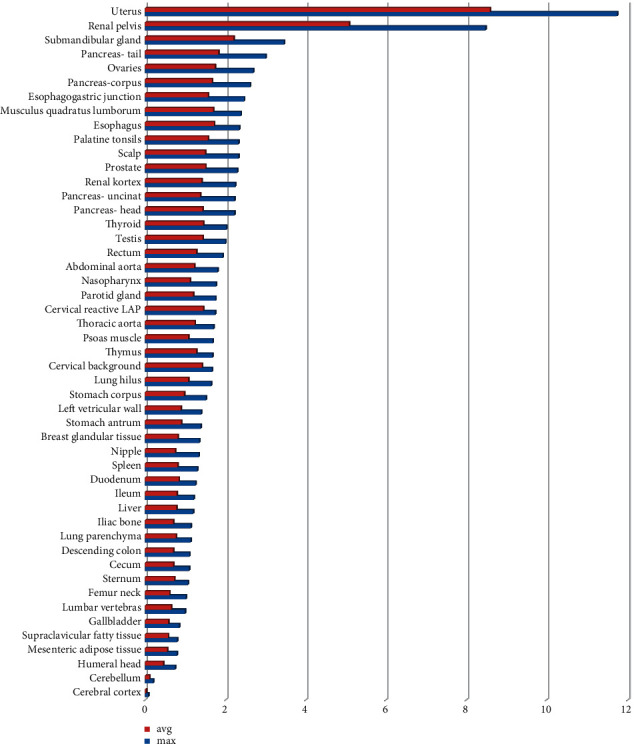
Maximum SUVmax and SUVavg of tissues and organs.

**Figure 2:**
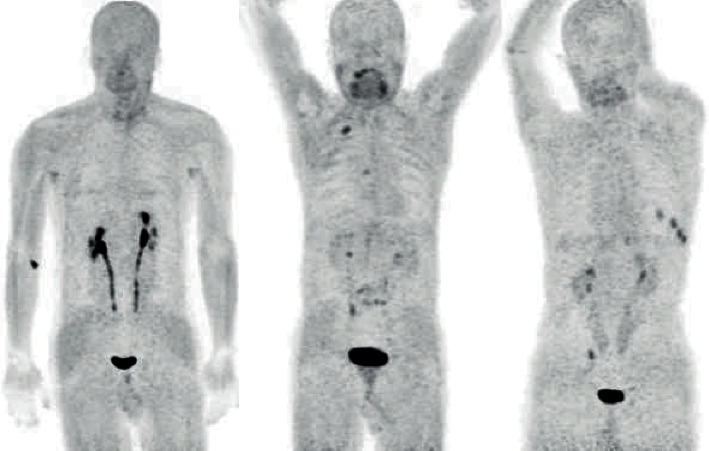
In the MIP images, renal excretion of FAPI-04 and urinary physiological uptake in the bladder are observed.

The accumulation of ^68^Ga-FAPI in normal organs showed variations (Figure 1). The conventional maximum intensity projection images showed uptake of ^68^Ga-FAPI in the pelvicalyceal system and the urinary bladder with the excreted activity (Figure 2).

**Figure 3:**
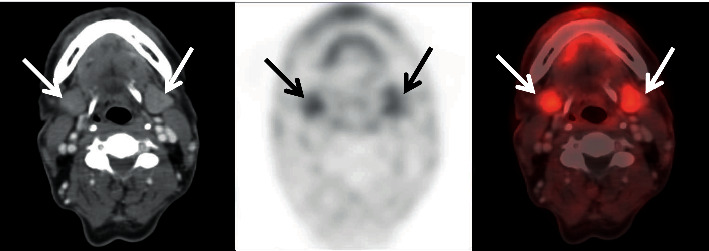
The FAPI-04 SUVmax level was 4.72 and the SUVavg level was 2.84 in the submandibular glands at axial CT, PET, and fusion images (arrows) of a 54-year-old male patient.

The median, mean, minimum, maximum, and standard derivation of the measured SUVs for all organs included are shown in Table 1. Moving from the vertex to the pelvis, the following observations can be made regarding the distribution of the radiopharmaceutical: the cerebral/cerebellar cortex exhibited no ^68^Ga-FAPI uptake, while the scalp showed low uptake. Low uptake was seen in the palatine tonsils, thyroid gland, and submandibular glands (Figure 3). In most patients, the uptake in the cervical and mediastinal lymph nodes observed located on CT was not higher than the cervical background activity which is obtained from regional major vascular structures. Low uptake was also observed in the lung parenchyma, esophagus, left ventricle walls, nipple, and glandular breast tissue.

**Figure 4:**
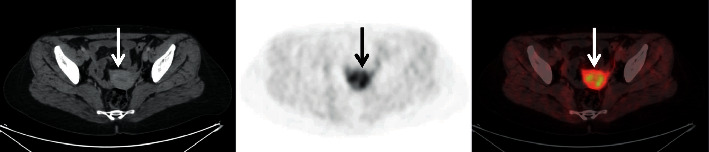
Physiologically intense FAPI-04 uptake was observed in the uterus (arrow) of a 38-year-old female patient without a history of hormonotherapy or oral contraceptive use, with an SUVmax level of 9.76 and an SUVavg level of 5.86.

In the abdominopelvic area, the pancreas exhibited low uptake, which was higher in the tail region. Low uptake was also observed in the renal cortex. Intense ^68^Ga-FAPI uptake was observed throughout the uterus, which was higher in the corpus (Figure 4). Six of 34 female patients had a history of hysterectomy, and the mean age of female patients with the uterus was 49.8 (32–72). The mean uterine SUVmax of those under the age of 50 (mean age: 41.7) was 15.8 and the average of those over 50 years (mean age: 60.6) was 12.3, and there was no statistically significant difference (*p*=0.28). There was no uptake of ^68^Ga-FAPI in the bone cortex and medulla.

## 4. Discussion

This study outlines the distribution pattern of ^68^Ga-FAPI and gives the range of normal SUVs in various tissues organs in the body.

High FAP expression has been demonstrated in the glandular cells of the gallbladder, urothelial cells in the bladder, endometrial glandular cells (low in stromal cells), smooth muscle cells, and glandular cells of the appendix in human studies [22]. Some studies have reported intense FAPI uptake in the uterus [19, 23]. Especially, the high uptake that we observed in the uterus is considered to stem from the endometrial glandular cells. Also, intense FAPI uptake in the uterus is independent from menopause in our study.

Moderate FAP expression has been demonstrated in the respiratory epithelium of the nasopharynx, bronchi and lungs, squamous epithelial cells of the esophagus, glandular cells of the rectum, seminiferous ductal cell of the testes (no expression in Leydig cells), glandular cells of the epididymis, glandular cells of the prostate, glandular cells of the fallopian tube, glandular and myoepithelial cells of the breast (no expression in adipose tissue), myocytes, and the skin (moderate in keratinocytes and low in melanocytes) [22].

The cerebral cortex, cerebellum, hippocampus, caudate nucleus, thyroid gland, salivary glands, colon, kidneys, seminal vesicle, vagina, cervix uteri, and lymph nodes, on the other hand, exhibit very low FAP expression [22]. Because the brain has lower FAP expression and FAPI-04 cannot cross the blood-brain barrier, we observed much lower activity in the brain parenchyma than the scalp in our study.

No FAP expression has been reported in the parathyroid gland, adrenal gland, oral mucosa, stomach, duodenum, small intestine, liver, pancreas, ovaries, adipose tissue, spleen, tonsils, and bone marrow [22]. Organs with high DPP4 expression include the parathyroid gland, salivary glands, duodenum, small intestine, liver, gallbladder, kidneys, seminal vesicles, and the prostate gland, while the bronchi and pancreas demonstrate moderate FAP expression [22]. The uptake on FAPI PET/CT in these organs, especially the pancreas and salivary glands, is considered to be due to the high similarity of DPP4 with FAPI.

In their study comparing different types of tumor and different radiotracer agents, Giesel et al. [13] ranked the body tissues in decreasing order of SUVmax 60 minutes after the FAPI-04 injection as the oral mucosa, thyroid gland, kidneys, blood pool, parotid gland, pancreas, muscle tissue, colon, myocardium, spleen, liver, spinal canal, lungs, adipose tissue, and brain. In our study, the tissues/organs exhibiting the highest SUVmax were, in decreasing order, the uterus, renal pelvis, submandibular gland, pancreas, ovaries, and esophagogastric junction. Shi et al. reported the SUVmax of the left ventricle wall, left atrium, and right ventricular wall in a patient with nonischemic chronic heart failure to be 2.60, 2.39, and 2.10, respectively [24]. In the current study, we observed the median SUVmax to be 1.39 in the left ventricular wall but did not separately measure the right ventricle and right atrium.

A previous study with FAPI-46 reported the organ with the highest SUVmax to be the liver [23]. On the other hand, we observed the highest SUVmax with FAPI-04 in the uterus.

In our study, we found physiological low-level uptake of activity at the thyroid, lung, liver, bone marrow, spleen, and parotid glands similar to the work of Wang et al. [19]; however, we found relatively higher SUVmax values in muscle tissue and pancreas level.

Our study has certain limitations. First is the limited sample size. Second, we could only include patients with a malignancy because the newly developed radiopharmaceutical agents cannot be studied in large healthy populations. However, this is probably the most statistically significant clinical study of ^68^Ga-FAPI-04 in healthy tissue due to the relatively large number of patients.

## 5. Conclusions

We determined the physiological uptake and SUVmax of ^68^Ga-FAPI-04 in different tissues and organs and created a guide for researchers. The fact that FAPI has a very low background uptake in most normal organs facilitates tumor detection and suggests that there will be fewer side effects in FAPI binding radionuclide treatments in provinces.

## Figures and Tables

**Table 1:** Median, mean, minimum, and maximum SUV values of tissues and organs.

	SUVmax		SUVavg		SUVavg
	Median	Min/max	Median	Min/max	Mean/SD
Uterine corpus	11.74	2.62–37.39	8.59	1.73–25.32	10.1 ± 6.06
Renal pelvis	8.48	1.38–99	5.08	0.99–62.99	9.9 ± 12.02
Uterine cervix	4.31	1.33–9.58	2.955	0.94–7.06	3.1 ± 1.42
Submandibular gland	3.46	1.28–8.95	2.2	0.78–5.61	2.4 ± 1.02
Pancreatic tail	3	0.94–8.26	1.82	0.55–5.45	2 ± 1
Ovaries	2.69	0.3–5.87	1.735	0.14–3.55	1.9 ± 0.86
Pancreatic corpus	2.61	1.04–2.58	1.66	0.79–8.62	2.2 ± 1.52
Esophagogastric junction	2.46	1.08–3.79	1.56	0.69–2.86	1.5 ± 0.45
Quadratus lumborum muscle	2.38	0.79–7.73	1.69	0.58–4.13	1.7 ± 0.73
Esophagus	2.34	1.15–9.11	1.71	0.87–3.9	1.8 ± 0.59
Scalp	2.32	0.98–5.18	1.49	0.56–3.5	1.5 ± 0.52
Palatine tonsils	2.32	1.14–4.68	1.56	0.7–2.76	1.6 ± 0.49
Prostate	2.29	1.09–5.5	1.5	0.65–2.7	1.6 ± 0.67
Renal cortex	2.24	1.22–3.73	1.4	0.73–2.33	1.4 ± 0.37
Pancreatic head	2.22	1.18–10.13	1.425	0.74–6.38	1.8 ± 1.24
Thyroid	2.02	0.76–10.83	1.44	0.56–6.79	1.7 ± 1.19
Testis	2	1–3.07	1.43	0.72–1.87	1.3 ± 0.39
Rectum	1.93	0.74–4.96	1.27	0.44–3.02	1.3 ± 0.57
Abdominal aorta	1.8	0.85–3.04	1.22	0.59–2.1	1.2 ± 0.35
Nasopharynx	1.76	0.57–6.91	1.11	0.33–3.82	1.1 ± 0.51
Parotid gland	1.75	0.83–3.65	1.19	0.55–2.49	1.3 ± 0.41
Thoracic aorta	1.7	0.83–3.72	1.23	0.56–2.46	1.3 ± 0.41
Reactive lymph nodes (cervical)	1.74	0.98–6.88	1.44	0.85–4.24	1.5 ± 0.69
Psoas muscle	1.68	0.51–3.61	1.07	0.35–2.3	1.1 ± 0.43
Thymus	1.67	0.96/2.17	1.27	0.72/1.58	1.3 ± 0.53
Lung hilum	1.64	0.6–3.16	1.07	0.4–2.3	1.1 ± 0.4
Stomach corpus	1.51	0.41–3.93	0.97	0.23–2.28	1 ± 0.4
Left ventricular walls	1.39	0.6–2.59	0.89	0.41–1.77	0.9 ± 0.32
Stomach antrum	1.38	0.58–4.2	0.9	0.33–2.51	1 ± 0.41
Breast glandular tissue	1.345	0.58–5.28	0.815	0.33–3.55	1.2 ± 0.79
Nipple	1.33	0.42–4.97	0.75	0.11–2.82	1 ± 0.61
Spleen	1.295	0.64–2.81	0.81	0.34–1.95	0.9 ± 0.33
Duodenum	1.25	0.6–2.92	0.84	0.37–1.67	0.9 ± 0.31
Ileum	1.205	0.24–3.56	0.79	0.17–2.51	0.9 ± 0.47
Liver	1.19	0.47–2.91	0.78	0.31–1.64	0.8 ± 0.3
Iliac bone	1.135	0.57–2.12	0.7	0.34–1.31	0.7 ± 0.21
Lung parenchyma	1.13	0.37–2.71	0.77	0.26–1.63	0.8 ± 0.3
Descending colon	1.1	0.53–2.13	0.7	0.32–1.51	0.7 ± 0.28
Cecum	1.09	0.08–2.39	0.705	0.05–1.59	0.7 ± 0.28
Sternum	1.06	0.52–1.05	0.73	0.34–1.15	0.7 ± 0.17
Femur neck	1.01	0.42–2.21	0.61	0.29–1.16	0.6 ± 0.21
Lumbar vertebrae	0.99	0.51–2.2	0.65	0.28–1.87	0.7 ± 0.26
Gallbladder	0.85	0.42–1.73	0.58	0.24–1.31	0.6 ± 0.27
Mesenteric adipose tissue	0.795	0.26–1.67	0.55	0.16–1.17	0.6 ± 0.27
Humeral head	0.75	0.44–1.93	0.45	0.25–1.38	0.5 ± 0.24
Cerebellar cortex	0.2	0.05–0.9	0.11	0.03–0.3	0.1 ± 0.07
Cerebral cortex	0.08	0.01–0.27	0.03	0.01–0.18	0.0 ± 0.03
Primary tumors	12.61	1.65–50.44	8.39	1.23–29.63	9.5 ± 5.87

SUV: standard uptake value, SD: standard derivation, avg: average.

## Data Availability

The data used to support the findings of this study are available from the corresponding author upon request.
